# DNA Methylation and the HOXC6 Paradox in Prostate Cancer

**DOI:** 10.3390/cancers3043714

**Published:** 2011-09-27

**Authors:** Anna Vinarskaja, Masanori Yamanaka, Marc Ingenwerth, Wolfgang A. Schulz

**Affiliations:** Department of Urology, Heinrich Heine University, Moorenstr. 5, 40225 Düsseldorf, Germany; E-Mails: vifanna@t-online.de (A.V); chi016@ndmc.ac.jp (M.Y.); marc.ingenwerth@uni-duesseldorf.de (M.I.)

**Keywords:** prostate cancer, epigenetic silencing, DNA methylation, homeobox transcription factors, WNT signaling

## Abstract

Overexpression of the classical homeobox transcription factor HOXC6 is frequent in prostate cancers and correlates with adverse clinical parameters. Since surprisingly many HOXC6 target genes are downregulated in prostate cancer, it has been posited that oncogenic effects of HOXC6 in prostate cancer may be unmasked by concurrent epigenetic downregulation of target genes exerting tumor suppressive effects. To test this hypothesis, we have studied the expression of three HOXC6 target genes, *CNTN1* (encoding a cell adhesion protein), *DKK3* and *WIF1* (encoding WNT growth factor antagonists) as well as DNA methylation of *DKK3* and *WIF1*. HOXC6 upregulation and association with poor prognosis were confirmed in our tissue series. The three target genes were each significantly downregulated in cancer tissues and expression of each one correlated inversely with that of HOXC6. Cases with lower *WIF1* expression showed significantly earlier recurrence (p = 0.021), whereas no statistical significance was reached for *CNTN1* and *DKK3*. Hypermethylation of *DKK3* or *WIF1* gene promoters was observed in a subset of cancers with downregulated expression, but was often weak. Our data support the hypothesis that HOXC6 target genes exerting tumor-suppressive effects are epigenetically downregulated in prostate cancer, but DNA methylation appears to follow or bolster rather than to cause their transcriptional inactivation.

## 1. Introduction

Prostate cancer is distinguished by a profusion of epigenetic alterations, which include consistent hypermethylation of several genes, frequent hypermethylation of numerous others, genome-wide hypomethylation of repeat sequences, changes in histone modifications and altered expression of chromatin regulatory factors [1,2]. Some of these epigenetic changes, notably hypermethylation of genes like *GSTP1*, appear to be associated with earlier stages of tumor development, whereas others are rather associated with tumor progression. The latter changes include hypermethylation of additional genes, hypomethylation of retroelements and overexpression of the histone methyltransferase EZH2. Accordingly, there is considerable interest in exploiting epigenetic changes associated with early development for the detection of prostate cancer on the one hand and alterations associated with progression for classification, molecular staging and prognostic purposes on the other hand [3,4].

Among the prominent targets of epigenetic alterations in human cancers are classical HOX genes encoding transcription factors regulating cell fate and differentiation [5,6]. These genes are located in four clusters. Whereas normal prostate expresses predominantly posterior genes from the A and B clusters, genes from the C and D clusters become activated in cancer tissues [7-12]. Both the causes and consequences of cancer-associated changes in HOX gene expression are insufficiently understood. Nevertheless, it is now well established that individual classical HOX genes can act as oncogenes or tumor suppressors in various human cancers [6,13].

In the prostate, specifically, there is convincing evidence for an oncogenic function of HOXC6. Its mRNA and protein have been found to be strongly overexpressed in many prostate cancers compared to their low expression in benign tissues [7,10,14,15]. The degree of HOXC overexpression parallels several clinical parameters of tumor progression, including Gleason scores [10,14,16]. Analyses of genes affected by *HoxC6* knockout in murine prostates or by up- or downregulation of HOXC6 in human prostatic cells identified targets in the WNT and Notch signaling pathways as well as *BMP7, FGFR2* and *PDGFRA* [15]. These target genes are upregulated by HOXC6 and could plausibly mediate its effects on prostate cancer progression and metastasis. Curiously, however, about half of the genes positively regulated by HOXC6 in experimental models are actually downregulated in prostate cancers, including three genes encoding inhibitors of WNT signaling, *WIF1, DKK3* and *SFRP1* [15]. Moreno [17] has proposed an elegant explanation for this apparent discrepancy. According to this hypothesis, HOXC6 can activate both target genes promoting and preventing prostate cancer progression, but epigenetic inactivation of its tumor-suppressive targets would restrict its effect to cancer-promoting genes. Indeed, all three WNT inhibitor genes have been reported to be downregulated or hypermethylated in prostate cancer [18-26]. Therefore, DNA methylation of these genes may prevent their activation by HOXC6. However, this hypothesis has not been investigated explicitly by studying expression of HOXC6 together with methylation and expression of these target genes in prostatic tissue samples.

Here we report an expression analysis of *HOXC6* and three of its target genes in a well-characterized series of prostate cancer tissues. Our data confirm the reported correlation of *HOXC6* expression with clinical parameters of prostate cancer progression. As predicted, *WIF1* and *DKK3* downregulation was related to *HOXC6* overexpression. Both genes were hypermethylated in some prostate cancer samples, but their hypermethylation was not well correlated with downregulation. Likewise, a third HOXC6 target gene, *CNTN1*, was concordantly downregulated. Taken together, our data suggest that downregulation of *HOXC6* target genes are often accompanied by DNA methylation but can occur independently of this epigenetic modification.

## 2. Results and Discussion

### 2.1. Expression of HOXC6 in Prostate Cancer Tissues

**Figure 1.**
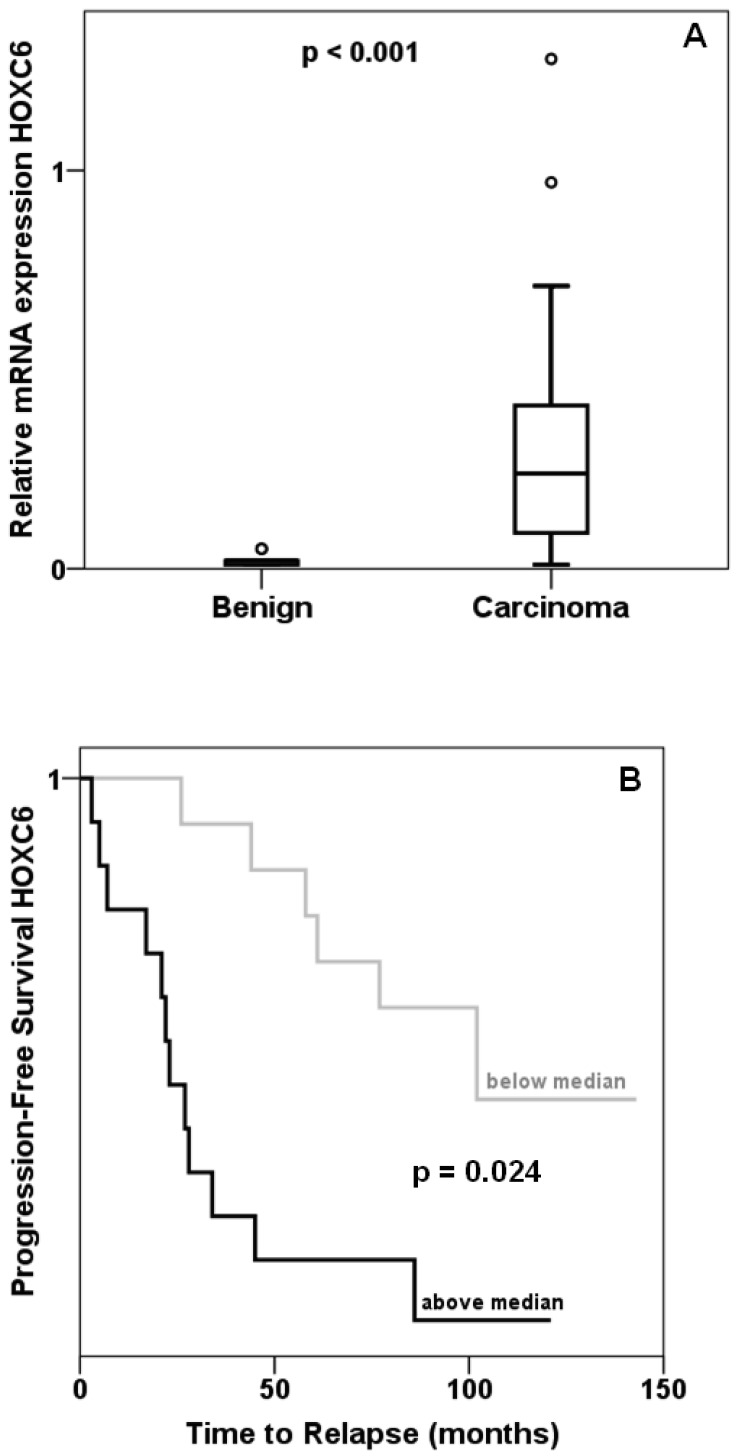
Expression of *HOXC6* in prostate cancer. (**A**) Expression of *HOXC6* mRNA as measured by quantitative RT-PCR in 45 prostate carcinoma and 13 benign prostate tissues; (**B**) Kaplan-Meier analysis of effect of *HOXC6* expression on biochemical recurrence.

Expression of HOXC6 was determined by quantitative RT-PCR in 45 prostate cancer and 13 benign tissues collected from prostatectomies. The majority of cancer tissues displayed—often grossly— elevated levels of HOXC6 mRNA resulting in an overall highly significant difference compared to benign tissues (Figure 1A). As reported by others, cancers with high *HOXC6* expression had significantly higher T stage, had more often spread to lymph nodes and were assigned higher Gleason scores. Expression of *MKI67* encoding the proliferation marker Ki67 was likewise enhanced in these cases (Mann-Whitney test: p = 0.004). Cancers with above median *HOXC6* expression recurred significantly (log-rank p = 0.024) earlier than cancers with below median expression (Figure 1B). These data confirm previous reports on frequent *HOXC6* overexpression in prostate cancer [7,10,14,15] and the association of increasing *HOXC6* overexpression with adverse clinical parameters.

### 2.2. Expression of Presumed HOXC6 Target Genes in Prostate Cancer Tissues

**Figure 2.**
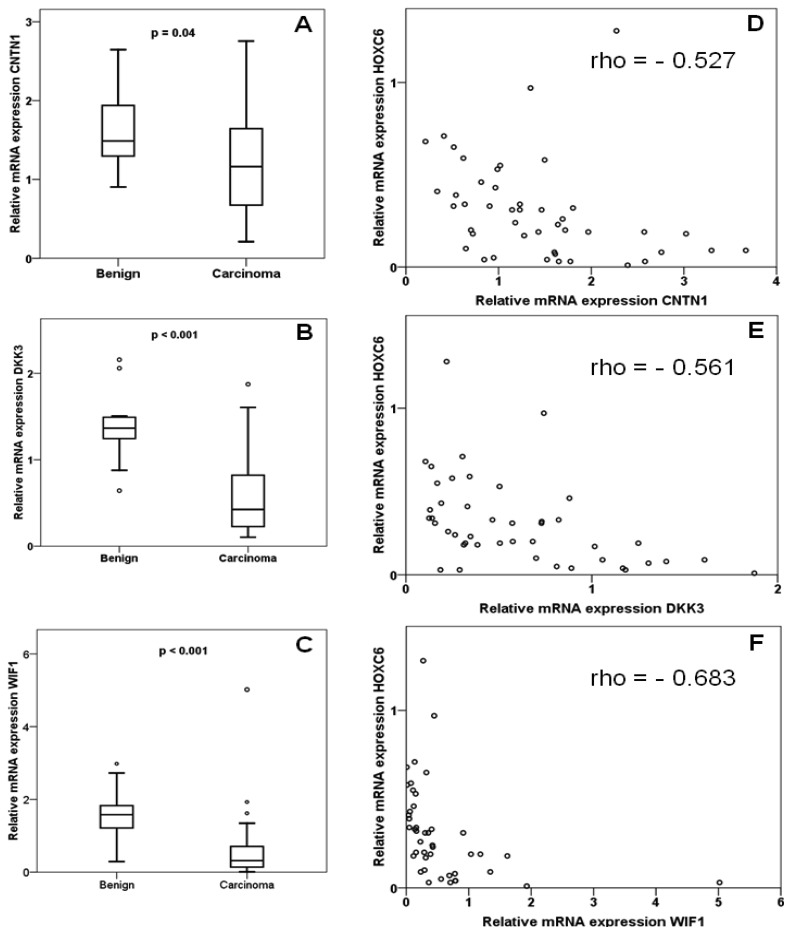
Expression of HOXC6 target genes in prostate cancer. (**A**) Expression of *CNTN1* mRNA as measured by quantitative RT-PCR in 45 prostate carcinoma and 13 benign prostate tissues; (**B**) Expression of *DKK3* mRNA in the same set of tissues; (**C**) Expression of WIF1 mRNA in the same set of tissues; (**D–F**) Plots of *CNTN1, DKK3* and *WIF1* expression against *HOXC6* expression. In each case, there was a highly significant (p < 0.001) inverse correlation.

**Figure 3.**
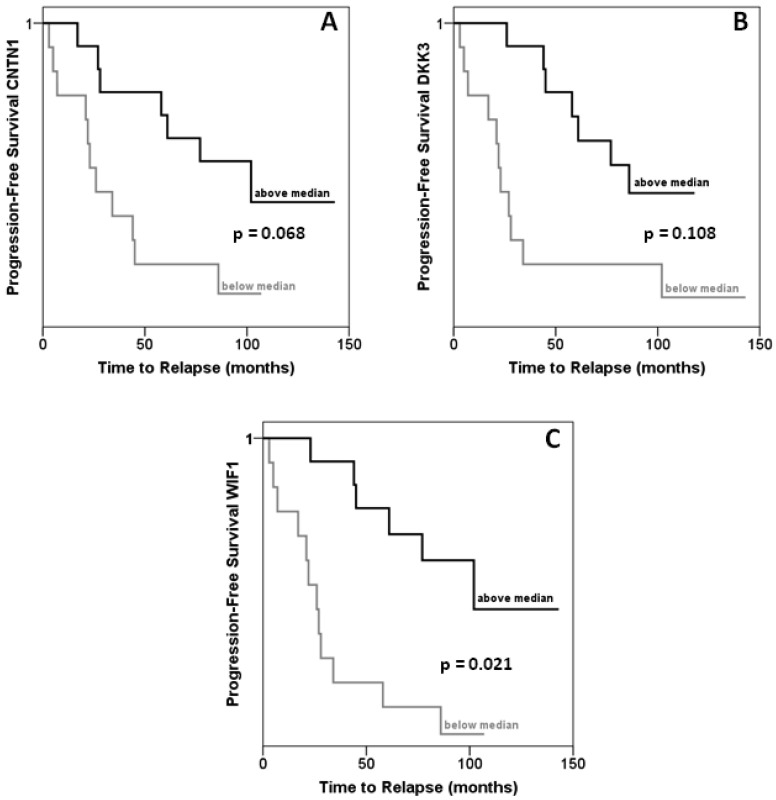
Relation of HOXC6 target gene expression to prostate cancer recurrence. Kaplan-Meier analysis of relation of *CNTN1* (**A**), *DKK3* (**B**) and *WIF1* (**C**) expression to progression-free survival, measured as biochemical recurrence. Samples were stratified by median for each gene.

In the same set of samples, expression of *DKK3, WIF1* and *CNTN1* was observed to be significantly decreased (Figures 2A–C). Expression of each gene correlated inversely with that of *HOXC6* in a statistically significant (each p < 0.001) manner (Figures 2D–F). Accordingly, expression of each target gene correlated significantly positively with that of each other, with Spearman rho coefficients between 0.4 and 0.6. Cases with lower than median expression of each target gene, *CNTN1, DKK3* or *WIF1*, showed earlier recurrence, but the association was only significant at the p < 0.05 level for *WIF1* (Figures 3A–C). In addition, low *WIF1* expression was significantly associated with lymph node involvement (p = 0.036) and higher Gleason scores (p = 0.026), but not with tumor stage (pT2 *vs.* pT3). Expression of *CNTN1* or *DKK3* was not significantly associated with any histopathological parameter in our series.

Our measurements confirm the reported downregulation of the WNT factor antagonists DKK3 and WIF1 in prostate cancer [18,21-23,25,26]. In addition, our data hint at an association of stronger WIF1 downregulation with worse prognosis. Most importantly, our study demonstrates for the first time explicitly that expression of certain target genes is inversely correlated with overexpression of HOXC6. This is also the first report on *CNTN1* in prostate cancer. The gene encodes a member of the contactin family which serves as a membrane receptor for chondroitin sulfate and regulates receptor tyrosine phosphatases. The function of contactin 1 has mainly been studied in neuronal and glial cells, where it regulates cell-cell and cell-substrate adhesion. Two studies in lung cancers and gliomas suggest that this function may also be relevant for cancer cell invasion and metastasis [27,28]. Investigations on the function of contactin 1 in prostate cancer and of the epigenetic regulation of its complex gene might therefore be rewarding.

### 2.3. Methylation of Presumed HOXC6 Target Genes in Prostate Cancer Tissues

**Figure 4.**
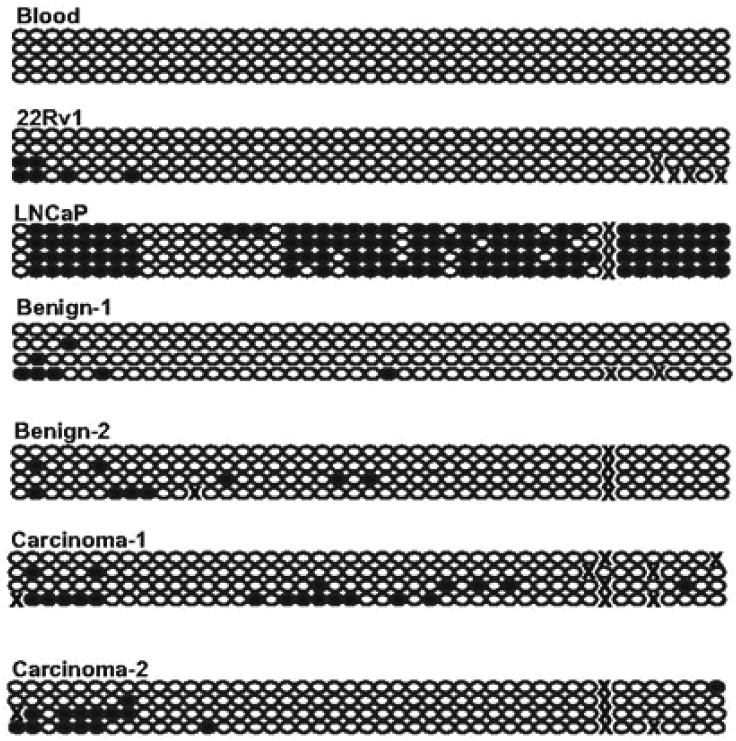
*WIF1* methylation in prostate cancer. Bisulfite sequencing analysis of *WIF1* promoter methylation in blood leukocytes, prostate cancer cell lines with high (22Rv1) and low (LNCaP) expression, benign and carcinoma prostate tissues. Each line represents one cloned PCR product, each circle represents one CpG site. Dark circles indicate methylated and light circles unmethylated sites. Some sites at the 3′-end of the sequence were difficult to read and are labeled by x.

Methylation of *DKK3* and *WIF1* promoter CpG-islands was analyzed by methylation-specific PCR as described in previous publications [21,29]. Methylation of *DKK3* was observed in 16 of the 92 carcinoma samples, but not in benign controls. It was significantly more frequent in cases with lymph node involvement and significantly less frequent in cases with Gleason score < 6 (χ^2^ test, p < 0.05). However, expression of *DKK3* was not significantly different between carcinoma samples with or without hypermethylation. *WIF1* methylation was more prevalent, being detectable in 31 of 92 carcinoma samples, but also in eight of 17 benign controls. The latter finding may relate to previous findings [18] suggesting that *WIF1* downregulation may commence at early stages of prostate cancer. As for *DKK3, WIF1* hypermethylation and the extent of downregulation of expression were not significantly related to each other. Because the bands obtained in the *WIF1* MS-PCR assay with the methylated-specific primers were often weak (except in LNCaP cells used as a positive control), bisulfite sequencing was conducted across the region interrogated by the assay in prostate tissue samples and controls (Figure 4). The analyzed part of the *WIF1* CpG-island was completely unmethylated in blood leukocytes, but was quite densely methylated in the prostate cancer cell line LNCaP which lacks *WIF1* expression. In contrast, only occasional sites were methylated in the expressing 22Rv1 line. In all benign and carcinoma tissues, only patchy and weak methylation was found, as suggested by the results of the MS-PCR assay. Of note, we have previously shown that in this set of prostate cancer tissues *GSTP1, EPB41L3* and several other genes are each hypermethylated at frequencies of 60%–80%, often displaying dense methylation [19,30]. Thus, *DKK3* and *WIF1* hypermethylation was much less widespread than downregulation and was often weak if it occurred.

## 3. Experimental Section

### 3.1. Patients and Tissue Samples

High quality RNA was prepared from 13 normal prostate tissues from cancer-carrying prostates and 45 carcinomas from patients aged 59–74 years undergoing total prostatectomy as described [19]. According to the IUAC 2007 TNM classification, tumors were staged as pT2 in 20, pT3 in 23 and pT4 in 2 cases. A Gleason score of 7 was detected in 26 tumors, < 7 in 13 tumors and > 7 in 6 tumors. None of the patients had distant metastases, but 11 cancers had spread to local lymph nodes. High quality DNA was available from 92 cancer tissues encompassing the specimens used for RNA analysis. Of these, 43 were staged as pT2 and 49 as pT3 or pT4. Sixteen patients had lymph node metastases, but none distant metastases. Each 27 carcinomas were assigned a Gleason score > 7 or < 7 and 38 a score of 7. The median follow-up period was 98 months. The study was approved by the ethics committee of the Heinrich Heine University medical faculty.

### 3.2. RNA Extraction and Quantitative RT-PCR

Total RNA was isolated from preconfluent cells using the RNeasy ^®^ Mini Kit (Qiagen, Hilden, Germany). Two μg RNA were reversed transcribed using SuperscriptII (Invitrogen, Karlsruhe, Germany) with oligo-dT primers according to the manufacturer's protocol. Quantitative real-time PCR for *CNTN1, DKK3, WIF1*, and the reference gene *TBP* was performed using SYBR-Green reaction mix (Qiagen) with 0.4 μM of each primer in an ABI Prism 7900HT instrument (Applied Biosystems, Darmstadt, Germany) with the following conditions: activation at 95 °C for 15 minutes followed by 45 cycles of denaturation at 94 °C for 15 s, elongation at 72 °C for 30 s and measuring at 88 °C for 15 s. Amplification lasted 30 s with temperatures at 55 °C for *CNTN1*, 57 °C for *DKK3* and *WIF1*, 61 °C for *TBP*. The quality of the amplification was assured by a melting curve established by incubation at 95 °C, 60 °C and 99 °C for 15 s each. *HOXC6* expression was measured by Taqman assays Hs00171690 mL (Applied Biosystems). Duplicate measurements gave < 10% difference. For each gene, a standard curve was constructed using a reference cell line with high expression and expression in the samples was expressed relative to this standard. The same procedure was performed for the *TBP* control, to which the measurements were then adjusted.

### 3.3. DNA Extraction and Methylation Analyses

High-quality DNA was extracted from tissues and prostate carcinoma cell lines as described [19]. The EZ DNA Methylation Kit (Zymo Research, Orange, CA, USA) was used for bisulfite conversion. PCRs were performed in a 50 μL reaction mixture consisting of 1 × buffer, 150 μM dNTPs, 15 pmol of each primer, 1 U Hotstar Taq polymerase, water and 2 μL bisulfite-converted DNA each. The following program was used: initial Taq activation at 94 °C for 15 min, followed denaturation at 95 °C for 30 s, annealing for 30 s, elongation at 72 °C for 45 s, with a final elongation for 10 min. MS-PCR amplification was performed for *DKK3* unmethylated/methylated for 36/38 cycles with annealing at 61/65 °C, for *WIF1* unmethylated/methylated MS-PCR amplification was performed for 34/36 cycles with annealing at 53 °C for both genes. Fully methylated and completely methylated controls were carried in each reaction as described [19]. For *WIF1* bisulfite sequencing PCR was conducted for 35 cycles with annealing at 60 °C. PCR products were examined by agarose gel electrophoresis. For bisulfite sequencing PCR products were cloned into the *E. coli* TOPO 10 vector (TOPO TA Cloning Kit, Invitrogen). Four clones from each sample were sequenced using standard methods. Primer sequences are compiled in Table 1.

### 3.4. Statistical Methods

Statistical calculations were performed using SPSS 19.0.

## 4. Conclusions

**Figure 5.**
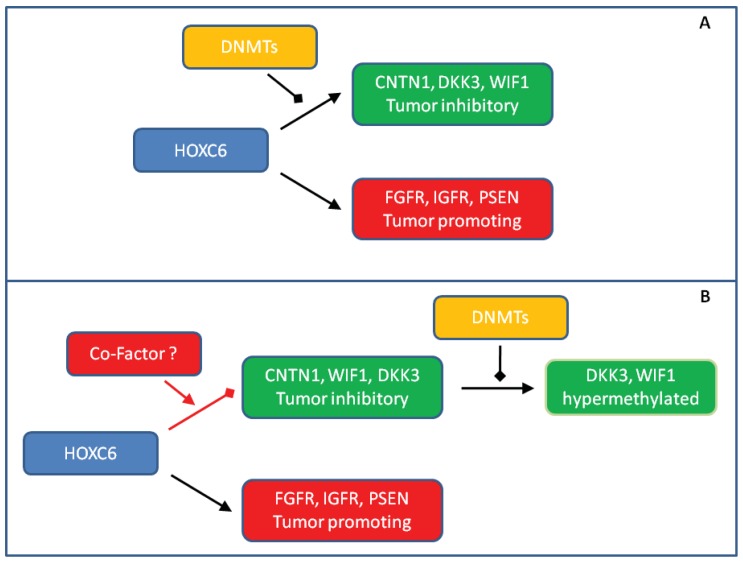
Hypotheses on the function of DNA methylation in inactivation of HOXC6 target genes in prostate cancer. (**A**) initial hypothesis based on ref. [14]; (**B**) hypothesis modified to explain the results of our study.

We observed striking *HOXC6* overexpression in our small, but well-characterized prostate cancer tissue series. Our findings underscore that the previously described strong association of *HOXC6* overexpression with adverse clinical parameters [7,10,14,15] is robust and deserves to be explored for the purposes of developing prognostic and molecular staging biomarkers. Our confirmation of these relationships accentuates the question of how HOXC6 contributes to oncogenesis and tumor progression in the prostate. Obviously, the answer is not trivial. Since HOXC6 is well-established as a transcription activator, it is most likely to act by regulating gene expression. Indeed, a range of positively regulated target genes have been defined by various experimental approaches. As reviewed by Moreno [17], among them are plausible candidates for promoting oncogenesis in the prostate, e.g., through WNT, BMP, FGF and NOTCH signaling pathways. However, it seems paradoxical that several genes activated by HOXC6 in experimental settings would be expected to counteract tumorigenesis *in vivo*, especially antagonists of WNT signaling like *DKK3* and *WIF1* [22,26,32]. These have indeed been reported to be downregulated or hypermethylated in prostate cancer in other studies [16,21,23]. One plausible explanation for the paradox is that genes antagonizing tumor development become hypermethylated in prostate by independent mechanisms and are no longer accessible to transcriptional activation by HOXC6 (Figure 5A).

Several results from the present study argue against this hypothesis in its most simple form. First, although we observed *DKK3* and *WIF1* hypermethylation in some cases, it was relatively weak and transcriptional downregulation was more generalized. Of note, we have previously found that hypermethylation of another HOXC6 target, *SFRP1*, is also relatively rare in our tissue series, whereas around 80% of the cases harbor hypermethylated GSTP1, as expected [19]. According results have been published by others [25]. Second, hypermethylation was not well correlated with transcriptional downregulation and was also observed in many benign adjacent tissues in the case of *WIF1*. These findings argue that DNA methylation is not the primary cause of transcriptional silencing of these genes, but may rather follow and bolster their initial downregulation by other epigenetic mechanisms (Figure 5B).

Third, we found the strongest downregulation of *CNTN1, DKK3* and *WIF1* in cases with high *HOXC6* expression suggesting that transcriptional silencing of these HOXC6 target genes might be elicited by the factor itself. This idea seems unlikely at first glance, since classical HOX factors act predominantly as transcriptional activators. There are however exceptions to the rule. For instance, HOX target genes may become repressed upon interactions with SMAD proteins that are activated by BMP signaling [33]. In order to explain our findings, we would like to propose the idea that the changed composition of transcription factors (and cofactors) in the nuclear milieu of prostate cancer cells may lead to a switch of HOXC6 function at some of its target genes (Figure 5B). An analogous case in prostate cancer is the altered target gene spectrum of the androgen receptor caused by changes in the expression of interacting transcription factors like HOXB13 and FOXA1 [34,35]. Accordingly, our data call for further detailed research on the HOXC6 paradox in prostate cancer which should yield important results for clinical application as well as insights into basic mechanisms of transcription.

## Figures and Tables

**Table 1.** Primers Used.

**Primer names**	**Forward 5′ → 3′**	**Reverse 5′ → 3′**	**Product size (bp)**	**Reference**
DKK3-MS-PCR (meth.)	GGG GCG GGC GGC GGG GC	ACA TCT CCG CTC TAC GCC CG	120	[29]
DKK3-MS-PCR (unmeth.)	TTA GGG GTG GGT GGT GGG GT	CTA CAT CTC CAC TCT ACA CCC A	125	[29]
DKK3-qRT-PCR	TTG CCA GCT TCC AGT ACA CC	TGC AGT GAC CCC AGA CAC A	105	self-designed
WIF1-MS-PCR (meth.)	CGT TTT ATT GGG CGT ATC GT	ACT AAC GCG AAC GAA ATA CGA	145	[29]
WIF1-MS-PCR (unmeth.)	GGG TGT TTT ATT GGG TGT ATT GT	AAA AAA ACT AAC ACA AAC AAA ATA CAA AC	154	[29]
WIF1-qRT-PCR	TAA TGG AGG GAC CTG TTT CTA CC	CCA TTT CGA CAG GGT TGT G	102	self-designed
WIF1-Bisulfite sequencing-PCR	GTT TTA GGG GTT TTT GAG TGT T	CAA CTC CCT CAA CCA AAA CTA	463	[31]
